# Phytoremediation technologies and their mechanism for removal of heavy metal from contaminated soil: An approach for a sustainable environment

**DOI:** 10.3389/fpls.2023.1076876

**Published:** 2023-01-27

**Authors:** Jitendra Kumar Sharma, Nitish Kumar, N. P. Singh, Anita Rani Santal

**Affiliations:** ^1^ Centre for Biotechnology, M. D. University, Rohtak, India; ^2^ Department of Biotechnology, Central University of South Bihar, Gaya, Bihar, India; ^3^ Department of Microbiology, M. D. University, Rohtak, India

**Keywords:** phytoremediation, heavy metals, soil contamination, crops, phytoextraction, metal-binding proteins

## Abstract

The contamination of soils with heavy metals and its associated hazardous effects are a thrust area of today’s research. Rapid industrialization, emissions from automobiles, agricultural inputs, improper disposal of waste, etc., are the major causes of soil contamination with heavy metals. These contaminants not only contaminate soil but also groundwater, reducing agricultural land and hence food quality. These contaminants enter the food chain and have a severe effect on human health. It is important to remove these contaminants from the soil. Various economic and ecological strategies are required to restore the soils contaminated with heavy metals. Phytoremediation is an emerging technology that is non-invasive, cost-effective, and aesthetically pleasing. Many metal-binding proteins (MBPs) of the plants are significantly involved in the phytoremediation of heavy metals; the MBPs include metallothioneins; phytochelatins; metalloenzymes; metal-activated enzymes; and many metal storage proteins, carrier proteins, and channel proteins. Plants are genetically modified to enhance their phytoremediation capacity. In *Arabidopsis*, the expression of the mercuric ion-binding protein in *Bacillus megaterium* improves the metal accumulation capacity. The phytoremediation efficiency of plants is also enhanced when assisted with microorganisms, biochar, and/or chemicals. Removing heavy metals from agricultural land without challenging food security is almost impossible. As a result, crop selections with the ability to sequester heavy metals and provide food security are in high demand. This paper summarizes the role of plant proteins and plant–microbe interaction in remediating soils contaminated with heavy metals. Biotechnological approaches or genetic engineering can also be used to tackle the problem of heavy metal contamination.

## Introduction

Metal ions at their higher concentration are toxic to plants, but they are necessary as trace elements. Many heavy metals (As, Cd, Cr, Cu, Hg, Ni, Pb, and Zn) are now hazardous to the environment globally and lead to a negative impact on human health. Due to their persistence in the environment for very long periods, such as for many hundreds to thousands of years, they negatively impact human and animal health (Alengebawy et al., 2021). Long-term exposure to heavy metals through the air, water, soil, and food causes various diseases like cancer, neurological effects, myocardial infarction, high blood pressure, skin lesions, organ system damage, urinary, reproductive, and respiratory systems (Rahimzadeh et al., 2017; Li et al., 2019). Lead (Pb) can persist in soil for more than 150–5,000 years and remains at high concentrations for up to 150 years after sludge application to the soil (Jabeen et al., 2009), whereas the biological half-life of cadmium (Cd) is approximately 10–30 years (Berglund et al., 2015). Removing heavy metals from the environment is very difficult because their degradation, like other pollutants, is not possible either biologically or chemically. Various technologies are adopted for *ex situ* and *in situ* heavy metal remediation of the contaminated soil. Some common technologies are chemical reduction, electrophoresis, excavation, pneumatic fracturing, soil washing, soil flushing, solidification, and nitrification (Dada et al., 2015). All of these traditional approaches are colloquially known as “pump and treat” and “dig and dump” techniques; however, these techniques are restricted to small areas and have limitations (Leung, 2004).

The conventional methods of removing pollutants from the environment are associated with numerous issues, such as partial removal, needing high energy, producing a significant amount of toxic sludge, being limited to a small area, and being costly (Li et al., 2019; Zamora-Ledezma et al., 2021). The economic burden of soil remediation by physical methods can be understood by the report of Salt et al. (1995). The phytoremediation of soil in 1 ac costs only approximately 60,000–1,000,000 US$, while physical remediation costs four-to-six times more to clean.

Over the last 10 years, a rapidly emerging, economically sound, and environmentally supportive alternative to traditional remediation practices has gained attention. This technique, known as “phytoremediation,” uses plants to clean up the environment as they can extract, accumulate, and depollute the substrate (soil, air, and water) from the contaminants through physical, chemical, or biological processes.

Several soil and plant factors influence phytoremediation efficiency, including chemical and physical soil properties, exudates from plants and microbes, metal bioavailability, and the plant ability to “uptake, accumulate, translocate, sequester, and detoxify metals” (Wang et al., 2020b). Bioremediation is economical as well as highly efficient; thus, these strategies have been proposed as an appealing alternative (Mejáre and Bülow, 2001). The application of plants and microorganisms either alone or in association to decontaminate heavy metal pollution has gained increasing attention. Many microorganisms, including fungi, mycorrhizal and non-mycorrhizal plants, and cultivated and wild plants, are tested in labs and the field for their ability to decontaminate metalliferous substrates in the environment (Tabrizi et al., 2015; Bahraminia et al., 2016; Yang et al., 2016; Yang et al., 2021; Antoniadis et al., 2021).

Understanding the mechanisms of how plants tolerate a specific metal is critical for increasing the number of plants that can be used for the phytoremediation of heavy metal–polluted sites. Various metal-binding proteins (MBPs) in the plants are involved in the absorption, accumulation, translocation, and detoxification of heavy metals and hence provide tolerance to the plants (Feki et al., 2021; Sharma et al., 2021). The MBPs include phytochelatins (PCs), metallothioneins (MTs), and transporter proteins [heavy-metal ATPase (HMA)] (Chaudhary et al., 2018; Mathur and Chauhan, 2020). This review focused on the different techniques used and the role of plant proteins to remediate soils contaminated with heavy metals. Biotechnological approaches or genetic engineering can also be used to tackle the problem of heavy metal contamination.

## Plants associated with the process of phytoremediation

The selection criteria for the plants used in phytoremediation are that they should be highly metal tolerant and have a short life cycle, broad distribution, large biomass, and a translocation factor (TF) greater than 1 (Mazumdar and Das, 2015). Some plant species are more suitable for phytoremediation than others. Two main factors are commonly applied for the assessment of the phytoremediation potential of a plant: bioconcentration factor (BCF) and TF. The shoot-to-root ratio of heavy metal and the root-to-soil ratio of heavy metal are defined as the TF and BCF. Plants with more than one TF and BCF (TF > 1 and BCF > 1) are expected to be used in phytoextraction (**Table 1**) (Li et al., 2022a).

**Table 1:** List of some plants used in the phytoremediation of different soil contaminants.

Heavy metals	Plants	BCF	TF	References
Zn (26.4 mg/kg) Cu (1.5 mg/kg) Ni (0.9 mg/kg)	*Sinapis arvensis;* *Brassica campestris;* *Brassica juncea*	7.5 6.47 6.71	1.49 - -	Drozdova et al., 2019
Cd, Zn (10–160 mg/kg)	*Tagetes erecta* L.	9.35 (Cd), 10.5 (Zn)	1.77 (Cd), 0.24 (Zn)	Madanan et al., 2021
Cu^2+^ (60–180 ppm)	*Helianthus annuus*	0.99	0.71	Mahardika et al., 2018
Pb, Zn (initial amount not provided)	*Pinus sylvestris;* *Quercus robur*	1.60 (Pb), 2.22 (Zn) 1.19 (Pb)	0.18 (Pb), 2.95 (Zn) 0.50 (Pb)	Andráš et al., 2016
Cd (5–100 mg/kg)	*Malva rotundifolia;* *Abelmoschus manihot*	3.31 3.67	7.37 1.21	Wu et al., 2018a; Wu et al., 2018b
Cd (5, 10, and 25 mg/kg)	*Pterocypsela laciniata*	4.55	3.73	Zhong et al., 2019
Cd (100 mg/kg)	*Lantana camara* L.	4.78	4.90	Liu et al., 2019
Ni (44.4 µg/L), Pb (114.6 µg/L)	*Typha angustifolia;* *Echhornia crassipus*	1.42 (Ni), 1.03 (Pb) 1.83 (Ni), 0.88 (Pb)	1.29 (Ni), 4.90 (Pb) 7.63 (Ni), 1.73 (Pb)	Pandey et al., 2019
Hg (230–6,320 ng/g)	*Plectramthus* sp.; *Clidemia* sp.; *Capsicum annuum;* *Phyllanthus niruri;* *Inga edulis*	0.33 0.36 0.83 0.59 0.28	1.73 1.43 1.19 1.12 1.21	Marrugo-Negrete et al., 2016
As (468.0, 442.0, and 304 mg/kg)	*Pteridium aquilinum;* *Corrigiola telephiifolia;* *Ludwigia erecta;* *Sacciolepis cymbiandra*	3.31 2.96 1.01 1.47	0.16 0.52 0.56 0.81	Onyia et al., 2021
Cr (100 mg/kg)	*Brachiaria mutica; Leptochloa fusca*	1.28 2.0	0.02 0.03	Ullah et al., 2021
Cr (50 mg/L)	*Typha angustifolia* L.; *Canna indica* L.; *Hydrocotyle umbellata* L.	2.6 10.9 37.8	1.03 1.17 0.191	Taufikurahman et al., 2019

## Mechanisms involved in phytoremediation of heavy metals

**Figure 1:**
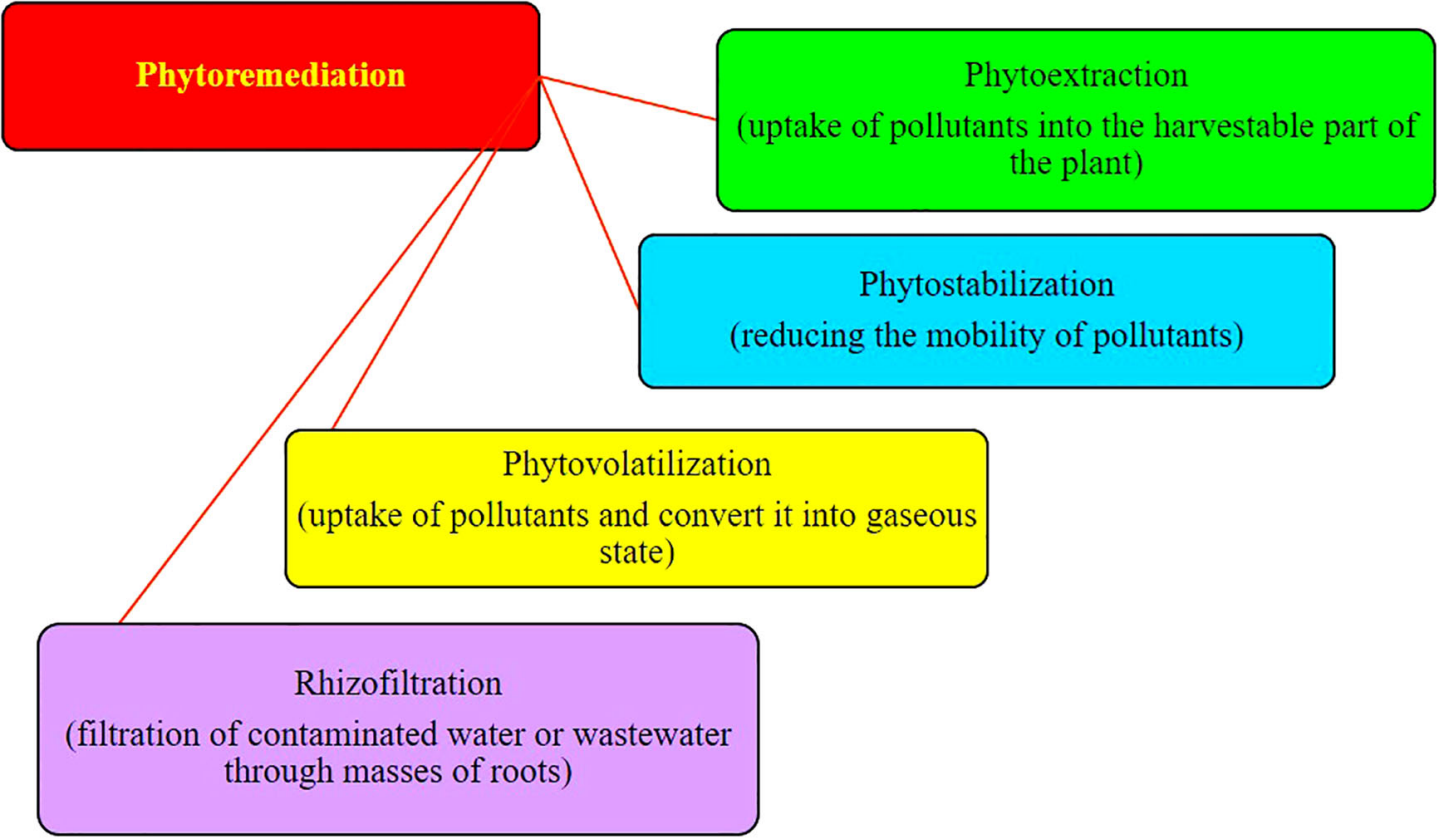
Various mechanisms involved in the phytoremediation of heavy metals.

The phytoremediation of heavy metal–contaminated soil includes any one mechanism or a combination of two or more phytoremediation mechanisms. The phytoremediation mechanisms mainly involved phytoextraction, phytostabilization, phytovolatilization, and rhizofiltration (**Figure 1**).

### Phytoextraction

Plants uptake pollutants from soil, water, or sediments by their roots and transfer them to the aboveground biomass where they accumulate, such as in shoots or other harvestable parts of the plant. This is known as phytoextraction (Singh and Santal, 2015; Sarwar et al., 2017; Yanitch et al., 2020). Plants that can accumulate heavy metals are cultivated on polluted sites for this reason, and the metal-enriched biomass above the ground is collected, resulting in the elimination of some portions of the soil pollutant. Since it is considerably easier to collect shoots than roots, metal transfer to shoots is an important physiological process. The most effective phytoremediation approach for heavy metal and metalloid removal from disturbed soils is phytoextraction. It is also the most commercially viable option. The efficacy of phytoextraction as a possible environmental cleaning solution is dependent on a variety of parameters, including heavy metal bioavailability, soil characteristics, heavy metal speciation, and the plant’s capacity to absorb metals and accumulate aboveground components (Yan et al., 2020). Approximately 450–500 different plants have been recognized as hyperaccumulators (Chaudhary et al., 2018). Plant species must have the following characteristics to be suitable for phytoextraction: (i) metal tolerance to harmful metals, (ii) the production of high biomass, and (iii) active accumulators of heavy metals in easily harvestable parts (Vangronsveld et al., 2009; Suman et al., 2018). The basic idea behind phytoextraction for polluted areas is to cultivate suitable plant species *in situ*, collect the heavy metal–containing biomass, and treat it to minimize its mass and size, which can be achieved through composting, compressing, dehydrating, and thermal decomposition. The resultant heavy metal–enriched biomass contains high levels of metal contaminants and, if economically feasible, is utilized for trace element reextraction or disposed of as carefully hazardous waste (McGrath et al., 2002; Sheoran et al., 2009; Suman et al., 2018). *Lemna valdiviana*, an American minute flowering plant, exhibits promising arsenic-bioaccumulating characteristics and can extract up to 82% of arsenic from contaminated water (Souza et al., 2019). *Bixa orellana*, used as an accumulator of the As(III) of Cr(VI), can accumulate 82.8% of Cr(VI) and 40.4% of As(III) of the initial amount of 3 and 6 ppm, respectively (Kumar et al., 2022b). Suthar et al. (2014) reported a significant phytoextraction potential of the maize (*Zea mays* L.). This potential was also increased with the addition of the metal chelating agent EDTA, which enhances more than 13-fold extraction of Pb and more than 3-fold extraction of Cd. Phytoaccumulation capacity for Cu contamination by three different lettuces (Romaine lettuce, Redina lettuce, and iceberg lettuce) was investigated by Shiyab (2018) who reported that Redina lettuce is a high accumulator of Cu. Accumulation in root tissue was 1.89 mg kg^−1^, while, in shoot tissue, it was 0.71 mg kg^−1^, which is relatively high among these three lettuces.

### Phytostabilization

Phytostabilization means establishing a plant covering the surface of polluted sites to limit the movement of contaminants within the vadose zone by root accumulation or immobilization inside the rhizosphere, therefore lessening off-site pollution. Transpiration and root development immobilize pollutants by a decrease in leaching, establishing aerobic conditions in the root zone, and the addition of organic substances to the substrate, which binds the pollutants (Bolan et al., 2011). The use of organic acid-producing metal-tolerant plant beneficial rhizobacteria, either alone or in combination with biogas residues, reduces Cd pollution in soil by stabilizing maize roots and limiting translocation to shoots. It also helps in improving maize biomass output, quality, and physiology. Furthermore, using poultry manure alone or in combination with biogas residues enhances Cd translocation to the shoot. Organic acid synthesis in maize root exudates is important for Cd stabilization in roots and shoots. Organic acid synthesis was raised in reaction to metal-tolerant plant-beneficial rhizobacteria and biogas residues, but poor production in response to poultry manure lowered Cd content in the root (Tahir et al., 2022). Mahdavian et al. (2022) reported that *Scariola orientalis* can be used as an efficient plant species in the phytoremediation (phytostabilization) of soils polluted with Zn and Fe. The level of heavy metal Cd in the rice grain can be reduced to improve the food quality. Cd levels in harvested rice (*Oryza sativa* L.) grains dramatically decreased in the Cd-contaminated fields when rice was intercropped with the alligator flag (*Thalia dealbata*). This reduction was accomplished because the fine roots of the alligator flag absorb a high quantity of Cd from the rice’s rhizosphere soil, lowering the rice’s Cd intake (Wang et al., 2020a). The alligator flag is most likely the best phytostabilization plant for Cd cleanup. Although soil nutrients varied significantly in the alligator flag cropping systems, Cd concentration was the dominant factor limiting microbial biomass and community structure. Intercropping *T. dealbata* with rice can be successfully used in the remediation of mild Cd contamination while simultaneously securely producing rice (Wang et al., 2020a). Naturally growing *Tetraena qataranse* plants in Qatar can accumulate heavy metal contaminants such as Cd, Cr, Cu, and Ni from the soil. This plant is suitable for animal fodder in arid areas (Usman et al., 2019). Two ecotypes of *Athyrium wardii*, one from a mining site and the other from a non-mining site, demonstrated different phytostabilization potentials for Cd from contaminated soils. The mining ecotype accumulated more Cd in roots, while the translocation of Cd to aerial parts was lower than in the non-mining ecotype. Furthermore, the use of humic compounds promotes the phytoremediation capacity of *A. wardii* root, particularly in the mining ecotype (Zhan et al., 2016).

### Phytovolatilization

Phytovolatilization refers to the use of plants to absorb heavy metal pollutants and transform them into volatile, less hazardous chemical species *via* transpiration. Some of the heavy metals, such as, Hg, and Se, may exist in the environment as gaseous species (Chandra et al., 2015). A small number of naturally occurring or genetically engineered plants, such as muskgrass (*Chara canescens*), Indian mustard (*Brassica juncea*), and *Arabidopsis thaliana*, have been shown to absorb heavy metals and transform them to gaseous forms within the plant before releasing them into the environment (Ghosh and Singh, 2005). *Arundo donax*, in association with the plant growth-promoting bacteria *Stenotrophomonas maltophilia* and *Agrobacterium*, can volatize approximately 75% of the initial amount of As (20 mgL^−1^). Approximately 25% remained in the sand, and only approximately 0.15% accumulated in the plant (Guarino et al., 2020). Arsenic exists in four different oxidative forms (−3, 0, + 3, and +5), but two commonly found species are arsenite (As^+3^) and arsenate (As^+5^). Formerly, it was believed that the microorganisms and enzymes reduced and methylated the arsenite and arsenate within the plants. Two As species, trimethylated and dimethylated As, were easily evaporated from the plants’ aerial parts (Zhao et al., 2010). However, recent reports proved that there is no involvement of the plant in the methylation of As from mono- and dimethylated or inorganic form to volatile trimethylated As species, although these volatile species are taken up by the plants’ roots from the soil itself (Jia et al., 2012). Selenium contamination in soil is also a great threat to the environment because of its long half-life period of approximately 327,000 years. It can be removed by the process of phytovolatilization (Sharma et al., 2015). Like As, Se also exists in nature in five different oxidative states (−2, 0, + 2, +4, and +6). The common species of selenium that is found in nature is in the selenate form (+6), and soil plants take it by sulfate transporters. In the plant, various biochemical processes and enzymes are involved in the conversion of inorganic Se to volatile (CH_3_)_2_Se (Sharma et al., 2015). Dimethyl diselenide [(CH_3_)_2_Se], dimethyl selenone [(CH_3_)_2_SeO_2_], dimethyl selenylsulfide [(CH_3_)_2_SeS], and methaneselenol (CH_3_Se) are also released by plants from the soil (Terry et al., 2000; Sharma et al., 2015; Chen et al., 2019). The phytovolatilization process also removes the neurodegenerative heavy metal Hg. The methylated form of Hg is a severe threat to humankind because of its biological magnification in the food chain (Kumar et al., 2017). Plants involved in the phytoremediation of Hg take it from the soil *via* their roots and translocate it to the aerial part of the plant *via* their vascular system, where it is then transpired. The enzymes of the plant transform Hg into a volatile form (Sharma et al., 2015).

### Rhizofiltration/hydraulic control

Rhizofiltration, or hydraulic control, is the method based on plant roots’ capacity to absorb and sequester metal pollutants from the water. Using this mechanism of phytoremediation, cleaning out metals such as Cd, Cr, Cu, Ni, Pb, and V and radionuclides (U, Cs, Sr) is possible (Jabeen et al., 2009; Singh and Santal, 2015). Long-rooted trees can absorb a large quantity of water, which was employed as a primary component in this procedure (Ahlfeld and Heidari, 1994). Long-rooted trees operate as pumps, drawing vast amounts of water from the subsurface water table (Muthusaravanan et al., 2018). As a consequence, contaminants in the water table are absorbed along with the water throughout this process. Root exudates such as citric acid and malic acid can scavenge or enhance the absorption, adsorption, or sedimentation of pollutants (Banerjee and Roychoudhury, 2022). *Z. mays* L. was evaluated (Benavides et al., 2018) and reported a 12% decrease in Hg, a 32% decrease in Pb, and a 30% decrease in Cr. The high potential of rhizofiltration is exhibited by the aquatic plant *Typha angustifolia*. It can uptake Cd and Zn 4,941.1–14,109.4 mg per plant and 14,039.3–59,360.8 mg per plant, respectively. *T. angustifolia* having a BCF value greater than 100 and a TF value less make it an excellent candidate for phytoremediation (Woraharn et al., 2021). Three very common aquatic plants *Azolla* (water fern), *Pistia* (water lettuce), and *Eichhornia* (water hyacinth) have different phytoremediation properties. *Pistia* has a good capacity for the phytoextraction and phytostabilization of As, Pb, and F, while, *Eichhornia* and *Azolla* effectively absorb Ni and Cu from the contaminated water. The TF of *Pistia* for fluoride is 5.0, making it an excellent hyperaccumulator of fluoride (Banerjee and Roychoudhury, 2022).

## Metal-binding proteins in plants

Several MBPs have been reported in plants, which include MTs; PCs; metalloenzymes (MEs); metal-activated enzymes; and many metal storage proteins, carrier proteins, and channel proteins (Memon and Schröder, 2009). Additionally, PCs are low-molecular-weight peptides having a high affinity for transition metals, synthesized by glutathione-derived metal-binding peptides (Clemens, 2006). MBPs are compounds that bind to metals such as Fe, Cr, Zn, As, Cd, Ni, and Pb (Mejáre and Bülow, 2001). Cysteine residues are abundant in naturally occurring heavy MBPs such as PCs and MTs. The presence of an increased chelating molecule in plant cells, such as MTs, which are cysteine-rich proteins, and PCs, is thought to be responsible for hyperaccumulating plants’ greater tolerance or resistance (cysteine- and glutathione-rich compounds) (Sharma et al., 2021). Plants possess different types of cadmium-binding proteins that have fewer cysteine residues (Yu et al., 2018). MBPs have often been introduced and/or overexpressed to improve bacteria and plants’ metal-binding capacity, tolerance, or accumulation. Plant PC biosynthesis has recently been changed to improve metal accumulation, while various peptides containing metal-binding amino acids (mainly histidine and cysteine residues) have been investigated in bacteria for greater heavy metal accumulation (Mejáre and Bülow, 2001). Some plant proteins involved in the phytoremediation of heavy metals are listed in **Table 2**.

**Table 2:** Plant proteins and respective genes in the phytoremediation of heavy metals.

Protein	Plant	Gene	Contaminant	Reference
Natural resistance–Associated Macrophage Proteins	*Arabidopsis thaliana*	*AtNramp1, 3, 4*, and *6*	Cd	Zhang et al., 2020b
Natural resistance–associated macrophage proteins	*Oryza sativa*	*OsNramp1, 2*, and *5*	Cd	Sasaki et al., 2012; Chang et al., 2020; Chang et al., 2022
Natural resistance–associated macrophage proteins	*Hordeum vulgare*	*HvNramp5*	Cd	Wu et al., 2016; Kintlová et al., 2021
Rubber elongation factor (REF)	Sweet potato (*Ipomoea batatas* L.), *Nicotiana tabacum*	*MuSI*	Cd, Cu	Seo et al., 2010; Kim et al., 2011
Phytochelatin synthase	*Sesbania rostrata*	*PCS1*	Cd and Zn	Li et al., 2009
Metallothionein and phytochelatin synthase	*Azolla pinnata* and *Azolla filiculoides*	*MT2* and *PCS1*	Ni, Zn, Cu, and Cd	Talebi et al., 2019
Phytochelatin synthase	*Nicotiana tabacum*	*PCS1*	Cd and As	Shukla et al., 2012
Phytochelatin synthase	Arabidopsis	*PCS1*	Cd and heavy metal(loid)s	Shukla et al., 2013
Phytochelatin synthase	*Ipomoea pescaprae*	*PCS*	Cd	Su et al., 2020
Fusion protein (bacterial mercury transporter MerC and a plant SNARE SYP121)	Arabidopsis	*MerC-SYP121*	Hg	Uraguchi et al., 2019
ABC-type xenobiotic transporter	*Arabidopsis thaliana* and *Populus tomentosa*	*PtABCC1*	Hg	Sun et al., 2018
Calmodulin-binding protein NtCBP4	*Nicotiana tabacum* and *Arabidopsis thaliana*	*NtCBP4 and AtCNGC1*	Pb	Kumar et al., 2022a
Na^+^/H^+^ antiporter	*Salvinia minima* Baker	*SmNhaD*	Pb	Leal-Alvarado et al., 2018
Glucocorticoid receptors and cytokinin-beta-glucosidase	*Nicotiana langsdorffii*	Glucocorticoid receptor gene *and rolC* gene	Cr	Del Bubba et al., 2013

### Phytochelatins

Plant PCs are cysteine-rich low-molecular-weight polypeptides that are synthesized enzymatically, and their formation is stimulated by the presence of heavy metals (Chia, 2021). The PCs are structurally associated with glutathione synthetase (GSH), and the common structural formula of the PCs is “(γ-Glu-Cys)n-Aa,” where n ranges between 2 and 11 and Aa is an amino acid at the C-terminal. Due to the high range of “n,” the structural species of the PCs are also high (Grill et al., 2007; Vershinina et al., 2022). The C-terminal ‘Aa’ is generally represented by Gly. However, in several plant families, C-terminal ‘Ala,’ ‘Glu,’ and ‘Ser’ isophytochelatins have been reported (Vershinina et al., 2022). PCs chelate heavy metals by using their thiol groups. The complexes of metals and PCs that are produced as a consequence are stored in vacuoles (Ovečka and Takáč, 2014). The phytoremediation capacity of the PCs is largely dependent on their polymerization. An aquatic plant, *L. minor*, was evaluated for its phytoremediation capacity. PC species with a higher degree of polymerization (PC4, PC6, and PC7) accumulated more Cd than PC species with a lower degree of polymerization (PC2 and PC3) (Török et al., 2015).

### Metallothionein

Plants have developed some adaptations to tackle metal ion concentrations’ increase in soil. An excessive amount of essential metal ions also causes toxicity similar to the non-essential metal ions; the foresaid mechanism provides metal tolerance as well as plays a significant role in the detoxification of excessive metal ions. MT was first discovered in animals than in plants; plant MTs have been discovered only approximately 30 years ago (Joshi et al., 2016). The superfamily of MT protein includes 15 families combined from animals, plants, fungi, and cyanobacteria (Joshi et al., 2016). The plant MTs are grouped into four distinct subfamilies: p1 (class 1), p2 (class 2), p3 (class 3), and pec (class 4). The MT1 gene from *Cicer arietinum* is part of the P1 subfamily, together with MT1a and MT1c from *A. thaliana*. On the other hand, the MT2 gene from *C. arietinum* is part of the P2 subfamily, along with MT2a and MT2b from *A. thaliana*. *Musa acuminate* and *A. thaliana* MT3 are both members of the P3 subfamily. Members of the ‘pec’ subfamily include *A. thaliana* MT4a (Ec-2) and MT4b (Ec-1), in addition to Ec-1 from *T. aestivum*. There are four different kinds of MT-encoding genes, and these genes may be found in *Arabidopsis*, rice, and sugarcane (Joshi et al., 2016).

### Transporter proteins

The transporter proteins of the plants can uptake, translocate and, sequester the heavy metals to provide tolerance to the plants and eventually remediate the contaminated soil. The transporter protein involved in the uptake of the heavy metals such as Cd is divided into several families based on the sequence similarity between them. The Cd transporter includes ZIP family transporter protein (Zn-regulated transporter protein and Fe-regulated transporter protein), metal tolerance proteins (MTPs), and natural resistance–associated macrophage proteins (NRAMPs) (Luo and Zhang, 2021). The HMA can transport heavy to the distant part of the plants e.g., the transportation of Cd from the root to the shoot (Mills et al., 2003; Wong and Cobbett, 2009). Many ions are stored in the vacuoles, and this storage can minimize the toxic effect of heavy metals. Various transporters are involved in the transportation of the free Cd and PC-Cd complex to the vacuoles. The vacuolar transporters are HMAs, NRAMPs, ATP-binding cassette transporters (ABCCs), and H^+^/cation exchangers (CAXs) (Lanquar et al., 2005; Park et al., 2012; Brunetti et al., 2015).

## Metal-binding proteins associated with different crops

Literature on the phytoremediation ability of cereal crops is sparingly available, although studies are mainly focused on the model plant *A. thaliana* and rice. In the present review, literature available on the other crops is also reviewed.

### Metal-binding proteins in rice

The elevated level of the heavy metal in the contaminated soil induced the expression of glutathione S-transferases (GSTs) and GSH in rice, which quenches the reactive molecules that induce the biosynthesis of the PCs. The PCs make a complex with the As, and the complex is sequestered into the vacuoles by ABCC1/ABCC2 transporters. Therefore, GST is involved in arsenic detoxification (Khan et al., 2018; Kumar and Trivedi, 2018; Tiwari et al., 2022). Localized near the root tip, OsNramp5 is a key transporter for Cd absorption in rice and a member of the NRAMP transporter family. In contrast, Cd is sequestered into root vacuoles by OsHMA3, a member of the HMA family. The OsHMA2, which is found in the root’s pericycle cells, has a role in mediating the translocation of Cd from the roots to the shoots (Wang et al., 2019).

### Metal-binding proteins in maize

Maize is established as a heavy metal accumulator; however, the detailed molecular mechanism is not amply known. The expression of the *ZmMTs* gene under heavy metal stresses (Cu, Cd, and Pb) regulated by the hormones and MT synthesis improves the growth and development of the maize plants (Gao et al., 2022). Jin et al. (2022) investigated the role of the PC synthase gene *ZmPCS1* of maize in Cd stress. The overexpression of this gene in the shoot and root of the maize plant prevented the toxic effect of the Cd and enhanced phytoremediation capacity. The expression of the PCs, MTs, and GSH in the root of the maize and barley plants increased when *Nocardiopsis lucentensis* (an actinomycete strain) was inoculated under As stress and enhanced the As-phytoremediation ability of both plants (AbdElgawad et al., 2021).

### Metal-binding proteins in wheat

The role of wheat in phytoremediation is sparingly studied; however, few studies reported an increase in the expression of the PC synthase (*TaPCS1*) gene under Cd stress and Pb stress (Repkina et al., 2019; Rahman et al., 2022). The application of Si to the wheat plants under As stress induces the level of PCs and MTs, which further reduces the translocation of As to the shoot by sequestrating it into the roots of wheat (Hossain et al., 2018).

### Metal-binding proteins in barley

Barley is the most abiotic stress-tolerant cereal crop against salinity stress, drought stress, and heat stress. It is also tolerant to some heavy metals like Cr, Zn, Cu, Cd, and Pb (Brunetti et al., 2012). A barley P1B-ATPase transports the important element Zn as well as the harmful pollutant Cd (Mills et al., 2012). The lipid-transfer protein from barley may play an important role in the phytoextraction of heavy metal ions from polluted soil. By using differential pulse polarography, Gorjanovi et al. (2004) evaluated the lipid-transfer protein-binding capacity to various metal ions and found that the protein has an affinity for Co (II) and Pb (II) but no affinity for Cd (II), Cu (II), Zn (II), and Cr (III) (Gorjanović et al., 2004). The expression of the *OsMT1e* gene for MT protein significantly enhances the Cd tolerance, detoxification, and accumulation of Cd ions (Rono et al., 2021).

## Advancements in the technologies used in the phytoremediation of heavy metals

The mechanism of the phytoremediation technique needs to be improvised with time to enhance efficiency as well as cost-effectiveness. Different approaches have been implied for the enhancement, and researchers are continuing to investigate new techniques; these include genetic engineering and phytoremediation assisted with biochar, chemicals, and microorganisms (Sarwar et al., 2017).

Biochar is an economical carbon-based material having porous nature, and, because of this, it has outstanding potency and adaptability in a variety of contexts (Brewer et al., 2014). Since it has an inherent benefiting soil-conditioning ability, which improves water-holding capacity, fertility, pH, nutrients, carbon sequestration, the activity of microorganisms, and the remediation of pollution, in recent years, there has been an increase in biochar research as a soil-ameliorating agent (Ennis et al., 2012). This is because biochar improves soil fertility. In its natural state, biochar has a pH range of 8–11 and its cation exchange capacity (CEC) ranges from 25 to 485 cmol(+) kg^1^. It has a large surface area (140–336 m^2^ g^1^), high porosity (0.0–1.32 cm^3^ g^1^), and a specific surface area that ranges from 10 to 400 m^2^ g^1^. The COOH, –CO–, –OH, and ester groups are all found on the surface of biochar, and their presence boosts the CEC and adsorption while simultaneously lowering the leachability of nutrients (Ahmad et al., 2014; Ghosh and Maiti, 2021). Biochar’s high pH organic material has the potential to minimize the bioavailability of heavy metals, which is a benefit to the environment. Because biochar remediation is not harmful to the environment and is cost-effective, its use for the amendment of soil that is polluted with HM has become more common. After applying biochar to soil, the average concentrations of accessible Cd, Pb, Cu, and Zn were found to have decreased by 52%, 46%, 29%, and 36%, respectively (Chen et al., 2018). According to Lu et al. (2017), adding bamboo, rice straw, and biochar at a concentration of 5% (w/w) reduced the amount of extractable Cd, Cu, Pb, and Zn found in polluted soil. Gong et al. (2019) reported the use of tea waste–derived biochar that can enhance the phytoremediation capacity of the plants. Biochar alleviates the toxicity induced by the Cd and improves plant growth. Biochar also promotes the enzyme-producing microorganisms in the Cd-contaminated sediments. Immobilization occurs as a result of the surface functional groups’ contact with the HMs. This interaction is responsible for 38%–42% of the total Pb^2+^ that is adsorbed. However, it is possible that biochar on its own will not be able to clean up a very polluted mine soil that has been contaminated with HM. Because of this, the interaction between biochar and phytoremediation has been observed to be successful. Various chemical compounds like EDTA, EGTA, and SDS have been added to soil or water to stimulate plant growth and increase phytoextraction. The accumulation of metals in various plant sections may be enhanced by chemical amendments without negatively impacting plant development. This makes one wonder how much and how precisely chemical additions need to be made to the soil to ensure optimal plant growth and metal phytoremediation. The application of flavonoids (rutin) to *Amaranthus hypochondriacus* under different Cd stress conditions reduces the cell membrane damage and provides tolerance to Cd toxicity. The application of ‘rutin’ can immobilize the Cd in the cell wall, and a less amount of Cd is transported to the vacuole; it also enhances the synthesis of GSH and conversion of GSH to PCs. The rutin enhances the phytoextraction capacity of Cd in *A. hypochondriacus* (219%–260%) (Kang et al., 2022). The application of the metal-chelating agent tetrasodium glutamate diacetate (GLDA) in combination with *Tagetes patula* L. improves the plant biomass and accumulation of cadmium (Cd). Plants can withstand Cd stress as well as can remediate contaminated soil; *T. patula* L. removes 12.9% of Cd from the contaminated agricultural land when GLDA is applied to the soil (Li et al., 2022b).

Although phytoremediation seems like a good way to get rid of metals in polluted areas, most plants have a negligible capacity to absorb metals. Therefore, plants with enhanced metal accumulation efficiency have been developed *via* genetic engineering. Plant species can be developed by gene transfer and gene editing focusing on metal absorption and transport processes involving PC and MT proteins (Ozyigit et al., 2021).

## Plant–microbe interaction

Growing in heavy metal-contaminated soil is never easy for plants; the first organ of the plant is the root, which gets exposed to the contaminants and faces severe stress from the surrounding metals and needs immediate help to withstand stress. Under stress conditions, the plant roots evolved an adaptive strategy, ‘cry for help,’ which attracted the beneficial microorganisms to help in minimizing damage (Rizaludin et al., 2021; Rolli et al., 2021). Plants are synthesizing many thousands of primary and secondary metabolites under different conditions and needs (Hartmann, 2004). These metabolites include volatile and soluble compounds that play important roles in recruiting plant growth–promoting microorganisms (PGPMs), which alleviate metal toxicity and promote plant growth (Ma et al., 2016; Rolfe et al., 2019). Under stress conditions, plants communicate with the different PGPMs by employing the root exudates of different quantities and compositions depending upon the type of stress (Rizaludin et al., 2021). The root exudates are very useful energy-rich nutrients for the soil microorganisms and are enriched with amino acids and organic acids; they also contain PCs, which bind the heavy metals (Mishra et al., 2017).

The recruited microorganisms by the above-mentioned strategies together with the root exudates free the heavy metals bound to the soil particle and make them available to the plants for phytoremediation (Khanna et al., 2022). Plant-associated microorganisms transform the heavy metals from a non-bioavailable form to a bioavailable form by various mechanisms like methylation, changing soil pH, redox processes, the production and secretion of siderophores, organic acids, and biosurfactants (Shah and Daverey, 2020; Sharma et al., 2021). The plant–microbial interaction between plant growth–promoting rhizobacterium (PGPR) *Variovorax paradoxus* 5C-2 and plant *Lotus edulis* and *L. ornithopodioides*. Under heavy metal stress, bacteria produce enzyme 1-aminocyclopropane-1-carboxylate deaminase, which enhances the uptake of Cd and promotes plant growth (Safronova et al., 2012). Siderophore formation, ACC-deaminase activity, and IAA production by the PGPRs *Pseudomonas reactans* EDP28 and *Chryseobacterium humi* ECP37 in association with maize plants improve the Cd uptake from the contaminated soil (Moreira et al., 2016).

Different biological processes by the microorganisms such as the chelation, complexation, immobilization, precipitation, solubilization, transformation, translocation, and volatilization of heavy metals change the mobility of the heavy metals, which improves heavy metal uptake and facilitates phytoremediation (Rajkumar et al., 2012; Singh et al., 2016; Shah and Daverey, 2020; Azhar et al., 2022). Microorganisms synthesize MBPs under heavy metal stress to increase the tolerance and accumulation of metals (Jach et al., 2022). In the plant–microbe association, MBPs significantly increase the accumulation of heavy metals and provide tolerance or resistance. Under stress conditions, plants and microbes adopt various mechanisms such as compartmentalization, the formation of complexes, exclusion, and the synthetization and secretion of MBPs like PCs and MTs (Sharma et al., 2021). Various plants and their associated microorganisms (bacteria/fungi) are given in **Table 3**.

**Table 3:** Plant–microbe interaction in various mechanisms of heavy metal phytoremediation.

Plant	Microorganisms	Heavy metal	Remediation %	References
*Zea mays*	*Serratia marcescens BacI56 and Pseudomonas* sp. *BacI38*	Hg	47.16% and 62.42%; endophytic; volatilization	Mello et al., 2020
*Medicago sativa*	*Bacillus subtilis*	Cd	139%; phytoextraction	Li et al., 2021
*Medicago sativa*	*Paenibacillus mucilaginosus*	Cu	55% and 76%; phytoextraction	Ju et al., 2019
*Solanum nigrum*	*Glomus versiforme*	Cd	90.0%; mycorrhizal; phytoextraction	Liu et al., 2015
*Brassica juncea*	*Aspergillus sydowii*	Cd and trichlorfon (pesticide)	10% and 4% phytoextraction and phytodegradation	Zhang et al., 2020a
*Phragmites communis*	*Simplicillium chinense*	Pb and Cd	29%–48.0%; mycorrhizal; biosorption	Jin et al., 2019
*Pelargonium hortorum*	*Aspergillus flavus and Microbacterium paraoxydans*	Pb	Two-to-fivefolds more uptake than control; Phytoextraction	Manzoor et al., 2021
*Vetiveria zizanioides* L.	*Bacillus cereus*	Cr(VI), Ni, Zn, Cu, Cd	130%–211% Cr(VI); 31%–40% (Ni); 30%–61% (Zn); 65%–178% (Cu); 84%–107% (Cd); phytostabilization	Nayak et al., 2018
*Sesbania sesban*	*Bacillus xiamenensis PM14*	Cr	56%; phytoextraction	Din et al., 2020
*Betula celtiberica*	*Rhodococcus erythropolis, Ensifer adhaerens, Variovorax paradoxus, and Phyllobacterium myrsinacearum*	As	16%–35%; endophytic; phytoextraction	Mesa et al., 2017

Genetically modified *Arabidopsis* showed enhanced accumulation capacity for certain heavy metals like mercury, cadmium, and lead. Hsieh et al. (2009) created a transgenic *Arabidopsis* that expresses the mercuric ion-binding protein (MerP) of *Bacillus megaterium* and reported the excellent metal-accumulating capability of *Arabidopsis*. Genetically modified rice plants were reported to accumulate more Cd in their root than shoot under the overexpression of the rice V-PPase (Cao et al., 2020). The coexpression of the wheat gene for the NHX antiporter and V-PPase proton pump diminishes the toxicity of copper in transgenic tobacco (Gouiaa and Khoudi, 2019). An artificially synthesized PC gene ‘*PPH6HIS’* was used to make the transgenic lines of the tobacco. The expression of the *PPH6HIS* gene in transgenic tobacco improves the accumulation capacity of Cd in the plant and provides resistance to the toxic effect of Cd on the plant (Vershinina et al., 2022).

## Conclusion and future prospects

The tolerance of heavy metal stress in plants is a defining characteristic of both their capacity to protect themselves and their efficient remediation systems. Utilizing model crops has provided several benefits in recent years for a better understanding of the biosynthesis, function, expression, and regulation of MT and PC. Even though several studies have shown that plant MTs play a significant role, we still have a long way to go before we can determine all of the tasks that MTs do. Their capacity to connect with a wide variety of metals, which results in a wide variety of functions, is shown by the enormous diversity that exists in the areas of metal binding in plant MTs compared to those in animal MTs. In *Arabidopsis*, MT-deficient mutants are not readily available, and it is probable that the members of the MT gene family have redundant functions. As a result, reliable information about the function of the gene is yet unavailable. Extensive research on “phytoremediation,” also known as the detoxification of contaminated surroundings, provides an overview of the potential of MT to combat stress tolerance. It is very necessary to exercise some level of control on the expression of MTs to boost the phytoremediation capabilities of plants. In addition, verifying their usefulness will need an understanding of how the accumulation and tolerance of metals are affected by the overexpression of these genes in certain organs. Microorganisms such as *Pseudomonas* sp., *Bacillus* sp., and *Aspergillus* and plants including *Brassica juncea, Solanum nigrum*, and *Z. mays* have a great ability to remediate an environment contaminated with heavy metals. Furthermore, several studies have revealed that plants and microorganisms work together to remove metal pollutants from the soil. The addition of plant growth-stimulating bacteria/fungi and metal-tolerant microorganisms improved the phytoremediation process considerably. As a result, selecting appropriate plant species and microorganisms can have a considerable impact on the outcome of phytoremediation. A comprehensive study of the root chemistry of metal-tolerant plants and microorganisms under stress conditions is much needed. How microorganisms and plants work together for the wellness of the metaorganism (resulting from the plant–microbe association) under particular stress conditions is required for further study for effective remediation of contaminated soil.

An emerging field of study and one with significant potential for commercial use is the creation of transgenic plants with an outstanding capacity to chelate certain metals and prevent the deleterious effects of these metals. In the future, the bioremediation of polluted places may benefit from the coordinated use of traditional breeding techniques in combination with molecular biology. Additionally, the identification of genes associated with metal tolerance *via* the use of genome sequencing might pave the way for the construction of transgenics with desirable characteristics that can be employed in phytoextraction technology. These findings, in association with considerable evolutionary research conducted across putative genes related to heavy metal tolerance, could provide some encouraging outcomes.

## Author contributions

All authors listed have made a substantial, direct, and intellectual contribution to the work and approved it for publication.
